# Preparation of Dual-Network Hydrogels and Their Application in Flexible Electronics

**DOI:** 10.3390/gels11120958

**Published:** 2025-11-28

**Authors:** Yang Yang, Jingna Jia, Chao Sun, Longbin Xu, Xinyu Li

**Affiliations:** 1 College of Engineering, Materials and Chemical Engineering, Yanbian University, Yanji 133002, China; 2025050044@ybu.edu.cn (Y.Y.); 0000008028@ybu.edu.cn (J.J.); 2 Department of Chemistry, College of Science, Yanbian University, Yanji 133002, China; 3 Department of Polymer Materials & Engineering, College of Engineering, Yanbian University, Yanji 133002, China; 4 Department of Physics, Jilin University, Changchun 130012, China; sunc344@jlu.edu.cn

**Keywords:** dual-network hydrogels, preparation, modification, flexible electronics, electronic devices

## Abstract

The rapid development of wearable technology has spurred considerable interest in hydrogels, which are hydrophilic three-dimensional polymer networks known for their remarkable flexibility. Nevertheless, their application in flexible electronics has been constrained by inferior mechanical and physical properties. As an emerging flexible material, dual-network hydrogels possess high mechanical strength, self-healing capability, excellent fatigue resistance, and electrical conductivity, showing great potential for use in flexible electronics. This article systematically reviews the design and performance optimization strategies of dual-network hydrogels. It reviews the advancements in their applications in flexible electronic devices, including bodily fluid biomarker sensors, flexible energy storage devices, health monitoring sensors, and physical motion sensors. The potential future challenges and opportunities for dual-network hydrogel materials are also discussed. This review aims to provide a theoretical foundation for developing next-generation dual-network hydrogels for flexible electronics and to promote their practical implementation in this field.

## 1. Introduction

With the proliferation of smart terminals and mobile internet, the development of wearable electronic devices urgently requires materials that combine high self-healing capability, mechanical properties, and physical performance [1]. Hydrogels are formed by the physical or chemical crosslinking of monomers containing hydrophilic functional groups [2,3]. Due to their excellent flexibility and ion transport capacity, they show significant advantages in the field of flexible electronics [4,5]. However, traditional hydrogels suffer from poor mechanical properties, fatigue resistance, low responsiveness, and inadequate self-healing performance [6], which greatly limit their application in flexible electronics. Therefore, it is necessary to design and develop hydrogel materials with higher mechanical properties, physical performance, self-healing capability, and sensitivity.

In recent years, dual-network hydrogels (DNHs, referring to a special hydrogel structure composed of two networks formed from two or more different materials interpenetrating to form a dual-network.), as novel materials, have attracted widespread attention in flexible electronic devices. They maintain the advantages of traditional hydrogels, such as high-water content and high viscoelasticity, while overcoming the drawbacks of low mechanical strength, poor conductivity, and poor fatigue resistance [7,8,9]. Ongoing research on DNHs has found that incorporating fillers (e.g., nanocellulose, carbon nanotubes, MXene) can effectively enhance the mechanical properties, conductivity, and sensitivity of hydrogels, addressing limitations such as poor stability, low electrical conductivity, and inferior mechanical performance. Research on the application of dual-network hydrogels modified dual-network hydrogels in the field of flexible wearable electronics is extensive yet fragmented. Therefore, a systematic review is needed to summarize the current state of research and facilitate further investigations into dual-network hydrogel materials for flexible wearable electronics.

This article outlines the preparation methods of DNHs, including physical crosslinking, chemical crosslinking, and physicochemical hybrid crosslinking. It proposes performance optimization strategies for DNHs from the perspective of adding modified reinforcing fillers, reviews their applications in flexible electronic devices, and finally provides an outlook on the future research and development of DNHs for flexible electronics.

## 2. Construction of Dual-Network Hydrogels

The preparation method of dual-network hydrogels is crucial for their properties and applications. Different methods impart different physicochemical properties, and optimizing the preparation process can develop hydrogels with superior performance to meet specific application needs. Based on the type of bonds formed between polymer chains during crosslinking, hydrogel preparation methods can be classified into physical crosslinking, chemical crosslinking, and physicochemical hybrid crosslinking.

### 2.1. Physical Crosslinking

Physical crosslinking refers to the process where polymer chains are connected through non-covalent interactions to form a three-dimensional network structure. Physical crosslinking mechanisms include hydrogen bonding [10], ionic interactions [11], and metal coordination [12], which often work synergistically. Physically cross-linked hydrogels enable gelation without the need for modifying polymer chains. When environmental or solution conditions change, such as temperature, pH, or ionic composition, these hydrogels can revert to biopolymer solutions. As a result, they are capable of actively reconfiguring themselves, exhibiting the ability to remodel and adapt to ambient conditions [13], Table 1 shows the method for preparing DNH by physical crosslinking.

#### 2.1.1. Hydrogen Bonding

Hydrogen bonding refers to the attractive, directional, and reversible interaction between a highly electronegative atom and a hydrogen atom [14]. Forming a network structure via hydrogen bonds is a common method for preparing DNHs, significantly enhancing properties such as mechanical performance, self-healing ability, and environmental adaptability.

**Figure 1:**
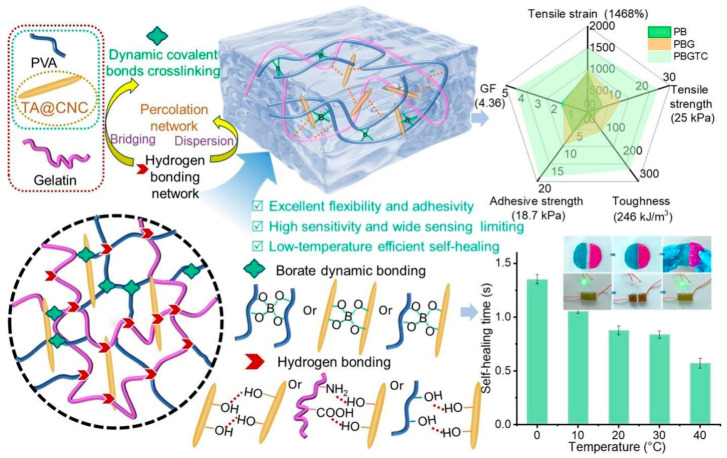
Schematic diagram of a dual-network hydrogel with a dual hydrogen-bonded multi-crosslinked interpenetrating network system [16]. Copyright 2025, Elsevier.

Introducing components with multiple hydrogen bonds into hydrogels can effectively enhance mechanical strength, self-healing capability, and environmental adaptability. Fang et al. [15] synthesized a dual hydrogen-bond network hydrogel (TiO_2_/MXene/PAA-PAM@AuNPs, TMPA) using a PAA-PAM hydrogel matrix with TiO_2_/MXene. The abundant -OH and -F functional groups on the MXene surface interacted with the PAA-PAM matrix via hydrogen bonding, promoting the formation of a dual hydrogen-bond network and significantly enhancing the mechanical properties, homogeneity, and adsorption performance of the hydrogel. Gu et al. [16] constructed a dual-network hydrogel (PBGTC) with a dual hydrogen-bonded multi-crosslinked interpenetrating network system using polyvinyl alcohol (PVA), borax, gelatin (Gel), and TA@CNC. As shown in Figure 1, strong hydrogen bond networks formed between PVA and Gel, and between tannic acid-coated CNCs (TA@CNC) and PVA/Gel. The resulting product exhibited improved tensile strength (25.54 kPa), toughness (246.09 kJ/m^3^), and self-healing efficiency (93.3%). Yuan et al. [17] prepared various hydrogen-bond crosslinked hydrogels by integrating natural microalgae frameworks with deep eutectic solvent (DES) synthesized from choline chloride and acrylic acid. An extensive hydrogen bond network formed between DES components and microalgae-derived functional groups, significantly enhancing the mechanical properties of the hydrogel: the fracture elongation increased from 167.54% to 635.42%, and the ionic conductivity increased from 0.12 S/m to 1.07 S/m.

As a weak chemical interaction, hydrogen bond networks can rapidly dissipate energy, contributing to the mechanical properties of hydrogels. However, due to their weak bond strength and low interaction density, the strength and tear resistance of hydrogels are limited, preventing them from absorbing large amounts of energy.

#### 2.1.2. Ionic Interactions

Ionic interactions are electrostatic attractions between ions of opposite charge [18]. Polyelectrolytes are polymers carrying a large number of charges, which can be positive (cationic polyelectrolytes) or negative (anionic polyelectrolytes). When multivalent ions (e.g., divalent or trivalent ions) with opposite charges are added to a polyelectrolyte solution, they undergo electrostatic interactions with the charged groups on the polyelectrolyte chains, forming ionic crosslinks [19].

Utilizing ionic interactions to prepare hydrogels is a current research focus. Jiang et al. [20] constructed a dual-network hydrogel (PHS-PA) using ionic interactions with phytic acid (PA), based on PVA and a zwitterionic polymer (SBMA-co-HEMA). The interaction between the negatively charged phosphate groups in PA and the positively charged quaternary ammonium ions in sulfobetaine methacrylate (SBMA) enhanced the ionic conductivity and ion transport of the hydrogel, and endowed it with excellent adhesion and adaptive properties.

**Figure 2:**
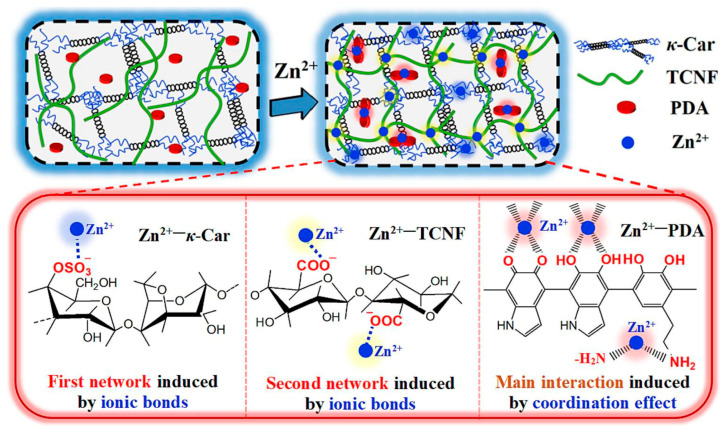
Schematic diagram of the construction of a novel κ-carrageenan dual-network hydrogel via ionic interactions [21]. Copyright 2023, Elsevier.

Strong ionic interactions between metal cations and hydrogel precursors can induce rapid gelation. Chen et al. [21] prepared a novel κ-carrageenan (κ-Car) dual-network hydrogel via ionic interactions by introducing TEMPO-oxidized cellulose nanofibrils (TCNF) and polydopamine (PDA) nanoparticles, as shown in Figure 2. Al^3+^, due to its trivalent nature, exhibits stronger electrostatic attraction and can form stronger bonds with the carboxylate groups of TCNF; Zn^2+^ binds via ionic interactions with the sulfate groups of κ-Car, the carboxylate groups of TCNF, and the catechol/amine groups of PDA. This not only promoted rapid gelation but also formed an interconnected porous structure, enhancing the mechanical properties and conductivity of the hydrogel. Wang et al. [22] synthesized a conductive hydrogel with a dual ionic network via ionic interactions using chitosan and PAA-co-AM. The trivalent Al^3+^ cations are primarily coordinated with the carboxyl groups of PAA-co-AM, consolidating the covalent network; after soaking chitosan molecules in sodium sulfate solution, they crosslinked with SO_4_^2−^ ions, forming numerous Ch-SO_4_^2−^ networks, providing the hydrogel with excellent tensile recovery, high strength, and toughness.

Although using ionic interactions to construct DNHs shows potential in enhancing mechanical properties and conferring conductivity, this method has limitations: it is often difficult to achieve both high strength and high toughness simultaneously, and the structure is prone to accumulated damage and incomplete self-recovery under large strain cycles. Furthermore, the environmental stability of these hydrogels remains insufficient; they are susceptible to ion loss, dehydration, or poor freeze–thaw stability due to changes in humidity and temperature, ultimately leading to issues such as low signal-to-noise ratio and sensitivity decay when used as flexible sensors, limiting their practical application.

#### 2.1.3. Metal Coordination

Compared to the simple, non-directional nature of ionic interactions (based on electrostatic attraction), metal coordination achieves directional bonding and spatial programming through orbital hybridization, combining covalent and ionic characteristics, whereas ionic interactions are inherently non-directional electrostatic attractions. Metal coordination is a versatile method for hydrogel crosslinking. By selecting different metal ions, ligands, and their corresponding concentrations, a wide range of elastic moduli can be achieved [23].

**Table 1:** Physical Crosslinking Preparation Methods for Dual-Network Hydrogels.

Preparation Method	Characteristic	Advantages	References
Hydrogen Bonding	Non-covalent Forces Reversible Directional	High self-healing efficiency (93.3%) Enhanced fracture elongation (635.42%) Excellent biocompatibility	[15,16,17]
Ionic Interactions	Electrostatic Interactions Reversible Non-directional Susceptible to ionic strength	Rapid Gelation Excellent Conductivity Strong Adhesion	[20,21,22]
Metal Coordination	High-strength Bonds Partially Reversible Directional Metal ion-sensitive	Excellent Conductivity (134.11 mS cm^−1^, 3.28 S m^−1^) High Tensile Strength (2.201 MPa, 0.92 MPa) Structural Stability	[24,25,26]

**Figure 3:**
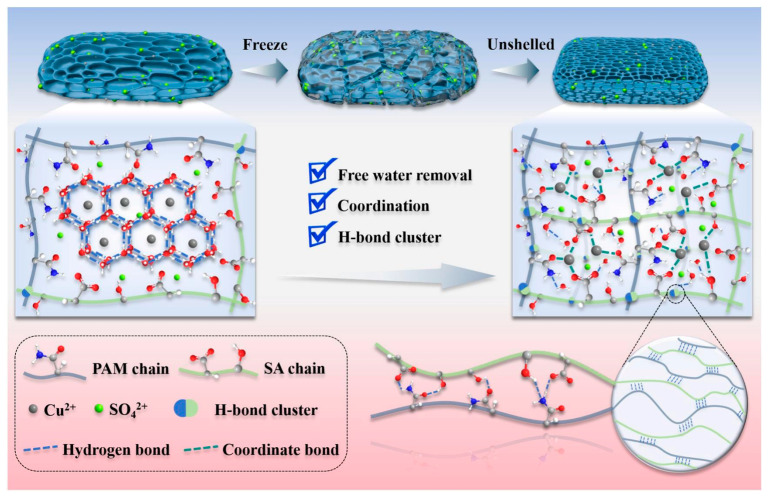
Schematic diagram of the mechanism for constructing a robust Cu/PAM/SA dual-network hydrogel via metal coordination [24]. Copyright 2025, Elsevier.

Metal ions play a key role in hydrogel formation, influencing their mechanical strength, self-healing properties, and conductivity. Wei et al. [24] prepared a dual-network hydrogel using a refreezing-assisted metal complexation (RAMC) strategy, as shown in Figure 3. Free water was removed in the form of ice shells, and functional groups on the molecular chains could form multiple coordinations with transition metals (Cu, Zn, Fe, Co, Ni), enhancing network structural strength and ionic conductivity. The resulting Cu/PAM/SA hydrogel exhibited a mechanical tensile strength of 2.201 MPa, high stretchability of nearly 250%, and a conductivity of 134.11 mS cm^−1^. Luo et al. [25] prepared an alkali lignin-polyvinyl alcohol-polyacrylic acid dual-network conductive hydrogel with high mechanical strength and good self-healing performance via metal coordination. The addition of Fe^3+^, forming metal-coordination bonds with PAA, significantly enhanced the mechanical strength, toughness, and self-healing performance of the hydrogel. Liu et al. [26] developed a tough, anti-swelling, highly conductive dual-network hydrogel via metal coordination. By adding Zr^4+^, which formed metal chelates with the carboxyl groups of acrylic acid (AA), the prepared hydrogel exhibited a tensile strength up to 0.92 MPa, toughness of 3.35 MJ m^−3^, conductivity of 3.28 S m^−1^, and excellent self-healing ability and anti-swelling capacity.

Metal coordination can serve as one of the crosslinking methods for DNHs, tuning hydrogel properties by forming coordination bonds between metal ions and functional groups on polymer chains [27]. However, while metal coordination bonds enhance strength and stiffness, they often sacrifice material ductility, toughness, and conductivity, making it challenging to simultaneously achieve high strength and high conductivity, or high toughness and rapid self-healing.

### 2.2. Chemical Crosslinking

Chemical crosslinking refers to the process where macromolecular chains are interconnected by chemical bonds under the action of light, heat, high-energy radiation, mechanical force, ultrasound, and crosslinking agents [28]. Chemically crosslinked hydrogels are held together by various covalent bonds, the nature of which depends on the polymer types present and the crosslinking mechanism. Chemical methods allow for more controllable and precise management of the crosslinking step [29]. This section mainly introduces free radical polymerization crosslinking, enzymatic crosslinking, photopolymerization crosslinking, and click chemistry crosslinking, Table 2 shows the method for preparing DNH by chemical crosslinking.

#### 2.2.1. Free Radical Polymerization Crosslinking

**Figure 4:**
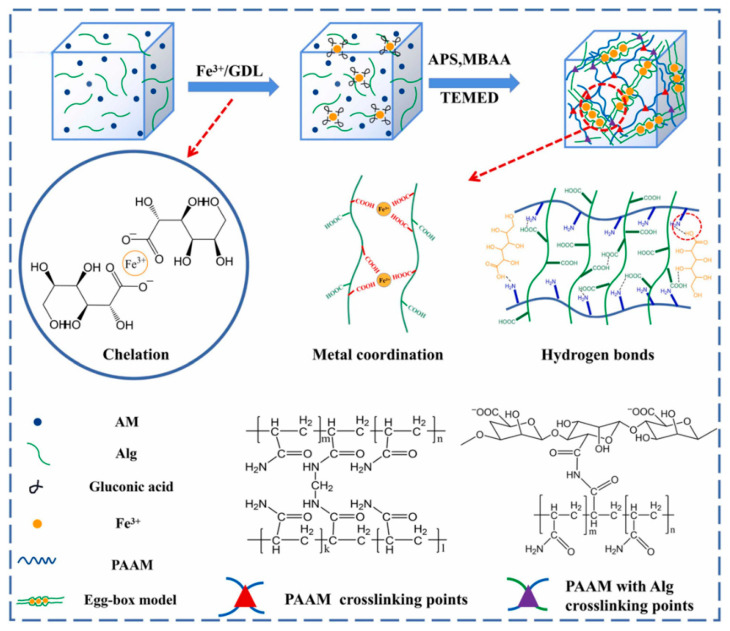
Schematic diagram of the synthesis route for constructing the Fe^3+^/Alg-PAAM dual-network hydrogel via free radical polymerization and the corresponding interactions within the dual network [33]. Copyright 2022, Elsevier.

Free radical polymerization is a chain polymerization reaction where monomers are added one by one to the active chain growth center under the action of a free radical initiator, rapidly forming high molecular weight polymers [30]. By adjusting reaction conditions and selecting suitable monomers, crosslinkers, and initiators, DNHs with specific properties can be prepared to meet application needs in flexible electronics [31]. Wang et al. [32] obtained a poly(N-isopropylacrylamide-co-acrylic acid)-Fe^3+^/chitosan-SO_4_^2−^ ionically crosslinked dual-network hydrogel (PICDN) via free radical copolymerization. PICDN exhibited significant temperature responsiveness, high sensitivity, and excellent mechanical properties, with a maximum tensile strength of 1.10 MPa and a fracture elongation of 225%. It also showed excellent self-recovery, fatigue resistance, and sensitivity under repeated cyclic strain. Zeng et al. [33] prepared a dual-network hydrogel (Fe^3+^/Alg-PAAM) based on sodium alginate, polyacrylamide (PAAM), and Fe^3+^ via free radical polymerization, as shown in Figure 4. PAAM and sodium alginate formed a covalently crosslinked network through free radical polymerization, endowing the product with a high storage modulus (2200 kPa), ultra-high stretchability (1800%), and strong adhesion to various substrates (61.8 kPa adhesion to glass). Kitiri et al. [34] prepared a dual-network hydrogel via free radical copolymerization using 2-(dimethylamino)ethyl methacrylate, lauryl methacrylate, and ethylene glycol dimethacrylate as raw materials. The resulting product exhibited pH responsiveness, nanophase separation, and high mechanical performance capable of withstanding high-stress environments.

Free radical polymerization is a powerful method for hydrogel synthesis, allowing the preparation of DNHs with specific properties by selecting appropriate monomers, crosslinkers, and initiators and precisely controlling reaction conditions [35]. However, the fast reaction rate and difficulty in precise control can lead to insufficient crosslinking density and inhomogeneous network structure.

#### 2.2.2. Enzymatic Crosslinking

Enzymatic crosslinking technology offers significant advantages in preparing starch hydrogels and other DNHs, as it avoids the use of traditional chemical initiators and organic solvents, providing efficient and mild reaction conditions [36,37]. By controlling physical parameters such as enzyme concentration, pH, and temperature, the reaction kinetics and gelation process can be regulated, enabling the preparation of DNHs with specific properties. Wang et al. [38] developed a dual-protein network hydrogel composed of bovine serum albumin (BSA) and silk fibroin (SF) using enzymatic crosslinking. This hydrogel exhibited excellent mechanical reversibility, frost resistance (−20 °C), highly stable conductivity, and a wide humidity monitoring range (11–85%). Wei et al. [39] prepared a PAM/DBS-COOH dual-network hydrogel composed of acrylamide (AM), crosslinker BIS, low molecular weight gelator DBS-COOH, and a glucose oxidase (GOx)-glucose-Fe^2+^ system using an enzyme-mediated method. GOx catalyzed glucose oxidation, producing H_2_O_2_ for initiating polymerization and gluconic acid for triggering self-assembly, significantly enhancing the mechanical properties and biocompatibility of the resulting product. Cernencu et al. [40] synthesized a dual-network hydrogel composed of methacrylated gelatin (GelMA), aminated pectin (AP), cellulose nanofibers (CNF), and two types of polyhedral oligomeric silsesquioxane (POSS0 and POSS1) via enzymatic catalysis. Transglutaminase (TGs) mediated the formation of isopeptide bonds between GelMA and AP, promoting protein-polysaccharide covalent bonds and significantly enhancing the mechanical properties, swelling behavior, and biocompatibility of the hydrogel material.

Enzymatic methods provide an efficient, mild, and controllable pathway for preparing DNHs. By selecting appropriate enzymes and reaction conditions, hydrogel materials with specific properties can be designed and synthesized. However, enzymes have high specificity for their substrates and are prone to inactivation under environmental conditions like temperature and pH, leading to demanding application conditions and poor stability.

#### 2.2.3. Photopolymerization Crosslinking

**Figure 5:**
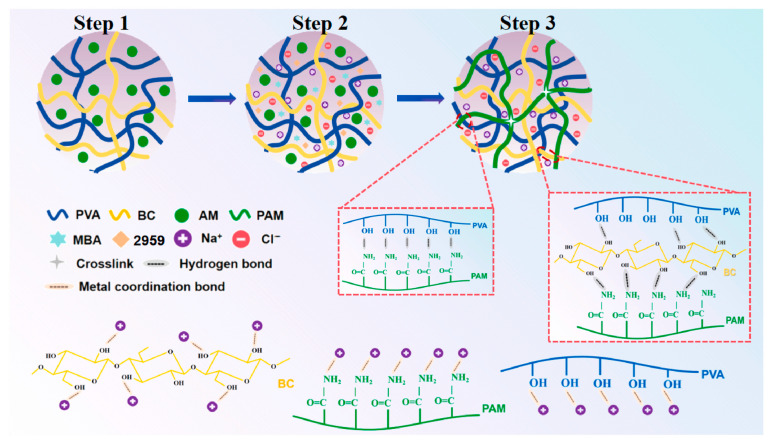
Construction of a dual-network structured hydrogel via photopolymerization: schematic diagram of the hydrogel formation mechanism [43]. Copyright 2025, Elsevier.

Photopolymerization crosslinking is a process that utilizes visible or ultraviolet light interacting with a photoinitiator to initiate chain polymerization and crosslinking of monomers, ultimately forming photocured oligomers with a network structure [41]. Photoinitiators play a key role in this process, initiating the polymerization reaction through photocatalytic action, enabling monomers to crosslink and form polymer networks with specific properties [42]. Lu et al. [43] synthesized a dual-network nanocomposite hydrogel (PAM/PVA/BC x-NaCl_y_ hydrogel) via in situ photopolymerization using AM, PVA, bacterial cellulose (BC), and NaCl as raw materials. The photoinitiator Irgacure 2959, under UV light, generated active radicals that initiated AM monomer polymerization to form the PAM network, resulting in a dual-network composite hydrogel with enhanced mechanical and conductive properties, as shown in Figure 5. Zhou et al. [44] synthesized a PDMAPS/P(DEAM-co-DMAM)/PCA-Na dual-network ionic conductive hydrogel (abbreviated LEC-Gel) via photopolymerization using an upper critical solution temperature (UCST) poly (ionic liquid) PDMAPS, a lower critical solution temperature (LCST) neutral polymer P(DEAM-co-DMAM), and the natural moisturizing factor sodium pyrrolidone carboxylate (PCA-Na). The resulting hydrogel exhibited ultra-high ductility (1500%), good fatigue resistance, and high sensitivity. Tang et al. [45] synthesized a PNIPAM/AM/MMA-HPMC dual-network hydrogel (abbreviated PNHMA) via photopolymerization using N-isopropylacrylamide (NIPAM), AM, methyl methacrylate (MMA), hydroxypropyl methylcellulose (HPMC), NaCl, and the ionic liquid [EMIM] BF_4_. Photocatalysis (i.e., UV irradiation at 365 nm wavelength) caused cleavage of the C=C bonds in NIPAM, AM, and MMA monomers, forming C-C bonds with the crosslinker MBA, endowing the resulting hydrogel material with low-temperature stability, high stretchability, high sensitivity, and thermoelectric responsiveness.

Photocatalytic synthesis of DNHs is a cutting-edge preparation method with advantages such as spatiotemporal controllability and mild conditions. However, many efficient photoinitiators (e.g., Irgacure 2959) may themselves be cytotoxic, and their residues or degradation products can limit the practical application of hydrogels. Moreover, the fast photopolymerization rate may lead to entanglement and less uniform distribution of crosslinking points compared to slower polymerization (e.g., thermal polymerization). This microstructural heterogeneity might affect the macroscopic toughness, fatigue resistance, and tear resistance of DNHs, preventing the full realization of their theoretical mechanical performance potential.

#### 2.2.4. Click Chemistry Crosslinking

Click chemistry crosslinking primarily involves the splicing of small molecules to efficiently achieve the chemical synthesis of different molecules [46]. Due to its simplicity, fast reaction rate, few byproducts, high yield, and good tolerance to other functional groups, it has attracted significant attention in hydrogel preparation [47]. The Schiff base reaction is an important type of click chemistry crosslinking, playing a key role in hydrogel preparation [48,49]. Schiff bases are imine compounds formed by the nucleophilic addition reaction between primary amines and aldehydes or ketones, possessing dynamic reversible characteristics and able to react under mild conditions without complex catalysts or protecting groups [50]. This section mainly introduces the Schiff base reaction as a major type of click chemistry crosslinking.

**Table 2:** Chemical Crosslinking Preparation Methods for Dual-Network Hydrogels.

Preparation Method	Characteristic	Advantages	References
Free Radical Polymerization Crosslinking	Irreversible Covalent Bonds High Bond Energy Stable Network Structure	Easily tunable reaction conditions customizable hydrogel functionalities.	[32,33,34]
Enzymatic Crosslinking	Irreversible/Dynamically Reversible Covalent Bonds Mild Reaction Conditions	Excellent Biocompatibility Controllable Reaction Kinetics	[38,39,40]
Photopolymerization Crosslinking	Irreversible Covalent Bonds Spatially and Temporally Controlled Polymerization	Rapid prototyping low-temperature processing	[43,44,45]
Click Chemistry Crosslinking	Dynamically Reversible Covalent Bonds Requires No Complex Catalyst Minimal Byproducts	Simple and Rapid Reaction Outstanding Self-Healing Capability (95.69% recovery within 25 min at room temperature)	[51,52,53]

**Figure 6:**
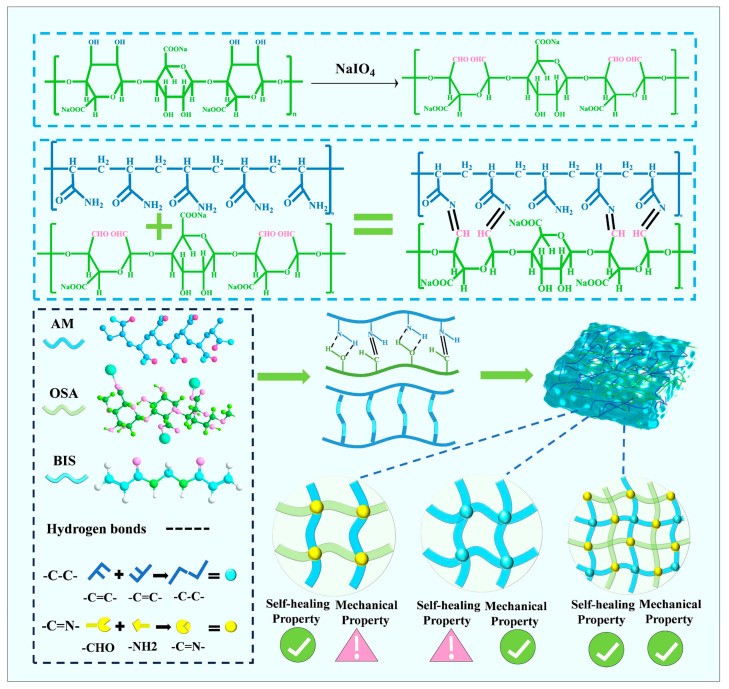
Preparation of (OSA/PAM) dual-network conductive hydrogel by click chemistry and its synthesis mechanism [52]. Copyright 2025, Elsevier.

The core role of Schiff base click chemistry lies in the formation of dynamic covalent bonds via the Schiff base reaction. Wan et al. [51] synthesized a dual-network gelatin/chitosan/emodin organohydrogel via Schiff base click chemistry using Gel, chitosan (CS), and emodin in Na_2_CO_3_ solution and a glycerol/water mixed solvent. The Schiff base reaction between the amino side chains of chitosan and the aldehyde groups in emodin molecules formed dynamic covalent bonds, significantly enhancing the mechanical properties of the resulting product, for instance, nearly tripling the tensile strength. Li et al. [52] synthesized an OSA/PAM dual-network hydrogel via Schiff base click chemistry using oxidized sodium alginate (OSA) and AM in phosphate-buffered saline (PBS), as shown in Figure 6. The aldehyde groups on the OSA chains and the amino groups on the PAM chains formed reversible imine bonds via dynamic Schiff base reactions, endowing the hydrogel material with ultra-high tensile strain (~770%) and rapid self-healing capability (95.69% recovery within 25 min at room temperature). Song et al. [53] constructed a fully dynamically crosslinked O-CMCS/PVA-based PBOC dual-network conductive hydrogel based on click chemistry reactions (such as Schiff base bonds) using oxidized pectin (O-PT), O-carboxymethyl chitosan (O-CMCS), PVA, borax, and carbon nanotubes (CNTs). The aldehyde groups on the O-PT side chains reacted with the NH_2_ groups of O-CMCS, forming reversible dynamic Schiff base bonds, endowing the hydrogel with excellent mechanical properties, high sensitivity, high adhesion, and self-healing performance.

The Schiff base reaction, as an important click chemistry crosslinking method, provides new ideas and means for the design and preparation of hydrogels. The introduction of dynamic covalent bonds can significantly improve the mechanical properties and self-healing ability of hydrogels. However, the dynamic imine bonds (-C=N-) formed by the Schiff base reaction are prone to reversible reactions in aqueous environments, especially hydrolyzing easily under acidic conditions, leading to unstable network structures.

### 2.3. Physico-Chemical Hybrid Crosslinking

**Figure 7:**
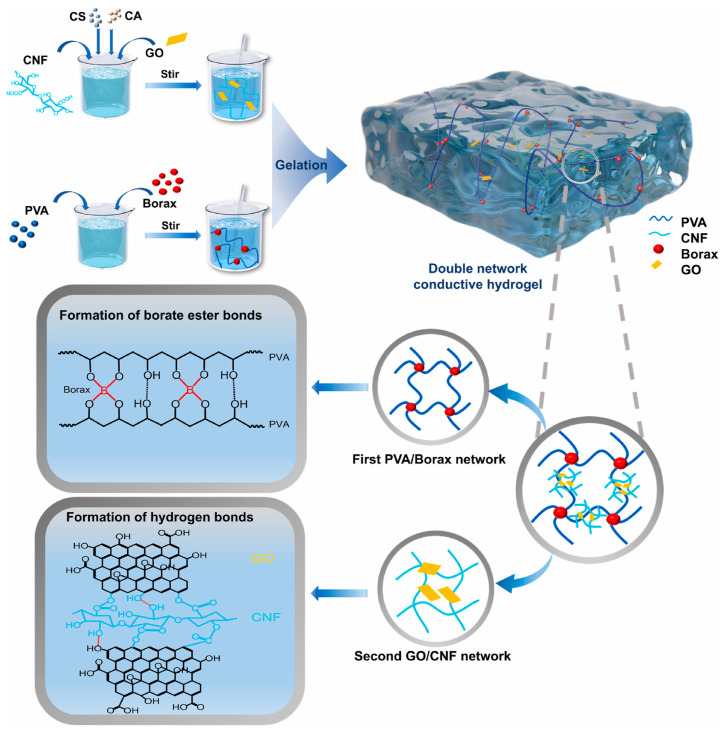
Schematic diagram of the preparation and sensor application of the PVA-Borax/GO-CNF dual-network hydrogel [56]. Copyright 2025, Elsevier.

Physico-chemical hybrid crosslinking is an effective strategy for preparing DNHs, combining the advantages of physical and chemical crosslinking to achieve excellent comprehensive properties. Through the synergistic effect of physical and chemical crosslinking, the mechanical strength, conductivity, self-healing ability, and environmental adaptability of hydrogels can be significantly enhanced, giving them broad application prospects in fields such as flexible electronic devices. Zhang et al. [54], through the synergistic effect of physical and chemical crosslinking, synthesized a PVA/PAA@PEGDMA/PEDOT: PSS dual-network conductive hydrogel (named PPPH) using PVA, AA, polyethylene glycol dimethacrylate (PEGDMA), and the conductive polymer PEDOT: PSS as raw materials. Chemical crosslinking was primarily achieved using PEGDMA as a multifunctional crosslinker, connecting short PAA chains into longer segments and elastic interchain bridges via covalent bonds, forming a uniform and highly interconnected network structure, and inhibiting microphase separation and structural defects. Physical crosslinking was achieved through hydrogen bonding entanglement between PVA molecules and the PAA network, as well as strong intermolecular forces via hydrogen bonding between the conductive polymer PEDOT: PSS and PAA. This significantly enhanced the mechanical strength and conductivity of the hydrogel. Wang et al. [55] adopted a physico-chemical hybrid crosslinking method to synthesize a dual-network hydrogel by introducing polypyrrole (PPy), using AM, N,N-dimethylacrylamide (DMAA), N-hydroxymethylacrylamide (NMA), and methyl cellulose (MC) as raw materials. Chemical crosslinking was primarily achieved via UV-induced crosslinking polymerization at 70 °C, using AM, DMAA, or NMA as monomers, and N,N′-methylenebisacrylamide (MBA) as the chemical crosslinker, ensuring the structural integrity of the first network. Physical crosslinking was achieved through multi-level hydrogen bonds, including the first-level hydrogen bonds (HB-1) formed between the amide groups of the AM derivatives and the hydroxyl groups of MC, the second-level hydrogen bonds (HB-2) formed between the imino groups of PPy and the amide groups of the AM derivatives, and the third-level hydrogen bonds (HB-3) formed between the imino groups of PPy and the hydroxyl groups of MC. This significantly enhanced the ductility, conductivity, strength, toughness, and self-healing capability of the hydrogel. Li et al. [56] developed a (PVA-Borax/GO-CNF) dual-network conductive hydrogel via a physico-chemical crosslinking strategy using PVA, borax, graphene oxide (GO), and CNF as raw materials, as shown in Figure 7. Chemical crosslinking was primarily achieved through the formation of dynamic borate ester bonds between PVA and borax, while GO and CNF formed ultra-rigid chemical crosslinks through covalent interactions and CH-π stacking. Physical crosslinking was achieved through freeze–thaw cycles to enhance the mechanical properties and stability of the hydrogel; hydrogen bonding between the amino groups of CS and the hydroxyl groups of PVA also promoted phase separation and increased porosity. This enabled the hydrogel to possess both high sensitivity and excellent mechanical properties.

Physico-chemical hybrid crosslinking provides an effective pathway for preparing high-performance DNHs. By deeply understanding the mechanisms of different crosslinking methods and combining advanced material design concepts and preparation techniques, novel hydrogel materials with excellent properties and broad application prospects can be developed.

## 3. Performance Optimization of Dual-Network Hydrogels

After decades of development, the various properties of DNHs have significantly improved compared to single-network hydrogels, but certain limitations remain. Ongoing exploration has found that performance can be effectively improved through modification methods such as adding fillers. This section mainly introduces modification methods involving the introduction of modified reinforcing fillers based on DNH materials, Table 3 shows the characteristics of different fillers and their performance improvement on DNH.

### 3.1. Nanocellulose

#### 3.1.1. Cellulose Nanocrystal (CNC)

Cellulose nanocrystal (CNC) is rod-shaped or needle-shaped cellulose crystals, also referred to as cellulose whiskers, cellulose nanowhiskers, or nanocrystalline cellulose. They are typically prepared via strong acid hydrolysis. During the hydrolysis process, the crystalline regions are preserved, resulting in crystals with high crystallinity. Consequently, CNC exhibits characteristics such as high crystallinity, high specific surface area, and high surface chemical reactivity. Due to their inherent outstanding chemical and physical properties, CNC is used as a reinforcing agent [57]. Incorporating CNC into a hydrogel matrix is a key strategy for enhancing the mechanical properties of hydrogels. Due to the cellulose backbone, CNC possesses a hierarchical structure. This structure creates numerous interaction sites between the polymer gel matrix and the surrounding CNC, which contributes to the improvement of mechanical properties [58]. The hydroxyl groups on CNC form hydrogen bonds with other polymer chains. In this way, CNC not only helps enhance mechanical properties such as stiffness and modulus but can also improve electrical conductivity by providing pathways for ion and electron transport.

**Figure 8:**
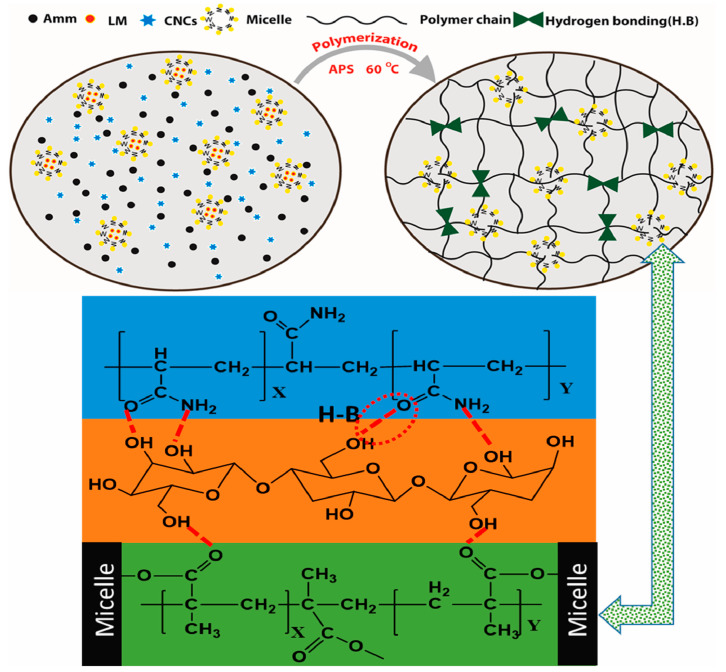
Schematic diagram of the polymerization process and the hydrogen bond between the polymer chain and the filler CNC [59]. Copyright 2023, Elsevier.

Khan et al. [59] prepared CNC-reinforced dual-network hydrogels composed of AM and lauryl methacrylate (LMC), as shown in Figure 8. The abundant surface hydroxyl groups of CNCs formed hydrogen bonds with the –NH_2_ groups in the AM polymer chains and the –C=O groups in both AM and LMC, reinforcing the hydrogel’s network structure. This led to improved mechanical properties, electrical conductivity, and endowed the resulting hydrogel material with low hysteresis energy, stability, high sensitivity, and a fast response time. Zhao et al. [60] fabricated CNC-reinforced PVA-based dual-network hydrogels. The abundant surface hydroxyl groups on CNC formed numerous hydrogen bonds with PVA chains, increasing the cross-linking density of the hydrogel. Furthermore, the CNC could undergo ordered alignment during stretching and, synergistically with PVA (a low birefringence material), form a photonic crystal structure, imparting significant optical anisotropy and stimulus-responsive behavior to the hydrogel. The tensile strength was substantially increased to 1.6 MPa, which is 6.7 times that of pure PVA hydrogel. Feng et al. [61] prepared CNC/PDA/ZP-reinforced dual-network hydrogels composed of PAA and CG. Utilizing hydrogen bonding interactions between PAA, CNC/PDA/ZP, and PDX, the synthesized product exhibited excellent anti-freezing performance (−35 °C), high sensitivity (GF = 2.97), and a fast response time (229.2 ms).

The abundant hydroxyl groups on the CNC surface can form numerous dynamic and reversible hydrogen bonds with polymer networks (such as the –NH_2_ and –C=O groups in PAM, or the –OH groups in PVA). These interactions work synergistically with the original network to establish a dual cross-linked network with a more effective energy dissipation mechanism. CNC significantly enhances the mechanical properties of hydrogels. Furthermore, due to their birefringence characteristics, CNCs confer rapid and visual stimulus-responsive capabilities to the hydrogels, in addition to imparting properties such as electrical conductivity, low hysteresis, high elastic recovery, and anti-fatigue characteristics.

#### 3.1.2. Cellulose Nanofibers (CNF)

Cellulose nanofibrils (CNF), also known as cellulose microfibrils, microfibrillated cellulose, or nanofibrillated cellulose, are primarily produced through mechanical methods. During mechanical processing, cellulose chains undergo longitudinal force-induced cleavage, enabling the extraction of nanocellulose [62]. This process results in less breakage of cellulose chains in the amorphous regions, endowing CNFs with a high aspect ratio and excellent flexibility. Unlike CNC, CNF is widely utilized as a nano-additive in hydrogels due to its renewability and abundant surface hydroxyl groups [63]. The intertwining of CNF with polymer chains restricts the free movement of chain segments, reducing slippage and deformation during stretching, thereby enhancing the toughness and ductility of the hydrogels.

**Figure 9:**
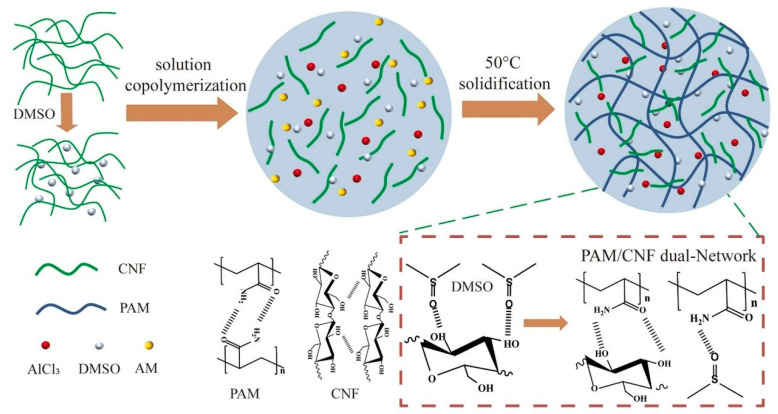
Schematic diagram of the preparation mechanism of PAM/CNF dual-network hydrogel [66]. Copyright 2025, Elsevier.

Goyal et al. [64] developed CNF-reinforced PAM/ALG1.5/3CNF-Fe^3+^ dual-network hydrogels synthesized from PAM and alginate (ALG). Under shear-induced alignment, CNF formed an oriented fibrous structure that enhanced the mechanical properties. The resulting hydrogel achieved a maximum tensile stress of 285 kPa and a toughness of 200 kJ/m^3^. Li et al. [65] prepared CNF-reinforced PAM/PVA dual-network conductive hydrogels using AM, PVA, and stearyl methacrylate (SMA). Extensive hydrogen bonding between CNF and the dual-network structure significantly improved the fracture stress, toughness, self-healing capability, sensitivity, and fatigue resistance of the hydrogel. Hong et al. [66] fabricated a PAM-based dual-network ionic conductive hydrogel reinforced with CNF, synthesized from AM, dimethyl sulfoxide (DMSO), and AlCl_3_, as illustrated in Figure 9. Through intermolecular hydrogen bonding and physical cross-linking, CNF enhanced the toughness, stretchability, self-healing ability, and thermal stability of the hydrogel.

As a versatile nanomaterial, CNF enables precise modulation of hydrogel properties—by adjusting their type, content, and modification methods—to meet the demands of various application scenarios. Specifically, CNF participate in both physical and chemical interactions with the two networks of the hydrogel: on one hand, they form dense hydrogen bonds with polymer chains (e.g., PAM, PVA), acting as reversible “sacrificial bonds” that effectively dissipate energy and restrict chain slippage, thereby facilitating efficient stress transfer; on the other hand, CNF serve as physical cross-linking points and a nano-reinforcing scaffold, bridging and stabilizing the entire dual-network structure and significantly increasing the cross-linking density. This dual mechanism leads to a qualitative improvement in the overall performance of the hydrogel, resulting in outstanding mechanical properties, excellent fatigue resistance, self-healing capability, and influence over swelling behavior via its three-dimensional network. Furthermore, CNF can guide ion distribution, thereby enhancing the electrical conductivity and sensing sensitivity of the hydrogel.

### 3.2. Carbon Nanotubes (CNTs)

Carbon nanotubes (CNTs) are one-dimensional nanomaterials composed of sp^2^-hybridized carbon atoms, forming a hexagonal honeycomb lattice structure similar to graphite, which endows them with excellent electrical conductivity [67]. CNTs exhibit extremely high strength and flexibility, with a tensile strength over 100 times that of steel while being significantly lighter [68]. Incorporating CNTs into hydrogels can significantly enhance their mechanical properties, electrical conductivity, and sensing performance [69,70].

**Table 3:** Comparison of Different Modified Fillers for Performance Optimization of Dual-Network Hydrogels.

Performance Optimization Filler Type	Specific Filler Types	Characteristics	Specific Performance Optimization	References
Nanocellulose	Cellulose Nanocrystals (CNC)	Rod-like/Needle-shaped High Crystallinity Hydroxyl-rich Surface	Enhancing tensile strength (1.6 MPa) high sensitivity (GF = 2.97) fast response time (229.2 ms) Optimizing the stability of the hydrogel network	[59,60,61]
	Cellulose Nanofibers (CNF)	High Aspect Ratio Good Flexibility Hydroxyl-rich Surface Renewable	Improving the toughness (200 kJ/m^3^) Enhancing the stretchability (285 kPa) self-healing ability thermal stability of hydrogels.	[64,65,66]
Carbon Materials	Carbon Nanotubes (CNTs)	High electrical conductivity ultrahigh tensile strength lightweight flexibility	Improving electrical conductivity enhancing mechanical strength (0.12 MPa) increasing sensing sensitivity (GF = 0.12 for 40–100% strain; GF = 0.24 for 100–250% strain)	[71,72,73]
2D Transition Metal Carbides/Nitrides	MXene	High electrical conductivity layered structure surface polar functional groups	Multi-functional integration Improving electrical conductivity Enhancing mechanical strength Enhancing EMI shielding performance Boosting the self-healing capability	[74,75,76]

**Figure 10:**
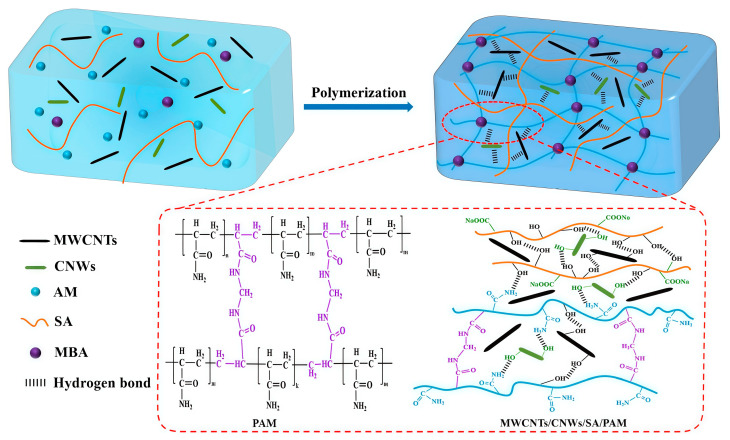
Schematic diagram of MWCNTs/CNMs/PAM/SA dual-network hydrogel [73]. Copyright 2025, Elsevier.

Han et al. [71] developed a dual-network hydrogel (TA-SA/P(AM-AMPS)-IL-OH-MWCNT) reinforced with ionic liquid and multi-walled carbon nanotubes (MWCNTs). The ionic liquid improved the dispersion of CNTs, and the excellent conductivity of the CNTs endowed the synthesized hydrogel with remarkable tensile strength (0.12 MPa), adhesive properties (0.039 MPa), and sensing performance (GF = 0.12 for 40–100% strain; GF = 0.24 for 100–250% strain). Luo et al. [72] prepared a PAM/gelatin/CMC dual-network hydrogel reinforced with CNTs, synthesized from carboxymethyl cellulose (CMC), aminated carbon nanotubes (NH_2_-CNTs), AM, and gelatin. The positively charged NH_2_-CNTs served as conductive enhancers and were uniformly dispersed within the hydrogel via electrostatic adsorption with the negatively charged CMC, effectively preventing agglomeration. This significantly improved the mechanical properties, broad-range sensitivity, and electrical conductivity of the hydrogel. Feng et al. [73] enhanced a CNWs/PAM/SA dual-network hydrogel, synthesized from cellulose nanowhiskers, AM, and SA, using MWCNTs, as shown in Figure 10. The MWCNTs filled the cross-linked network skeleton and pores of the hydrogel. Leveraging their high aspect ratio and metallic conductivity, they formed effective electron conduction pathways. This filling effect also helped maintain the stability of the hydrogel’s porous structure, significantly improving the mechanical properties, sensitivity, electrical conductivity, and stability of the material.

As a reinforcing material, CNTs significantly enhance the overall performance of dual-network hydrogels. The core mechanism lies in effectively addressing CNTs’ agglomeration through electrostatic interactions or with the aid of dispersing media like ionic liquids, enabling their uniform distribution within the hydrogel network. The uniformly dispersed CNTs act not only as nano-reinforcements, improving the mechanical strength and toughness of the hydrogel through interactions (e.g., hydrogen bonding) with polymer chains, but also establish efficient electron conduction pathways, thereby substantially increasing the electrical conductivity of the material. These structural advantages ultimately confer broad-range and highly sensitive strain-sensing capabilities to the hydrogels, making them ideal materials for preparing high-performance flexible sensors.

### 3.3. MXene

MXene, as a two-dimensional transition metal carbide/nitride, plays a significant role in enhancing the performance of dual-network hydrogels, particularly in the fields of strain sensing, mechanical strength, and electromagnetic interference (EMI) shielding. The incorporation of MXene can synergistically improve hydrogel properties through multiple mechanisms [74,75,76]. In strain sensing, MXene nanosheets can effectively slide and rearrange under small strains, maintaining electrical conductivity, whereas under large strains, the supramolecular interactions between MXene and polymer chains significantly increase electrical resistance, thereby enhancing strain sensitivity [77].

**Figure 11:**
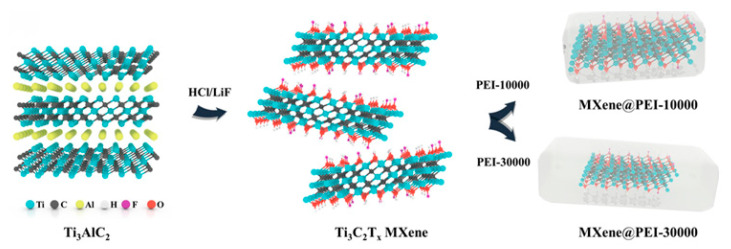
Schematic diagram of the preparation of MXP-x [76]. Copyright 2024, Elsevier.

Tian et al. [74] reinforced a PALCM dual-network hydrogel—synthesized from CNC, PAA, liquid metal (LM), and LiCl—using Ti_3_C_2_T_x_ MXene. The Ti_3_C_2_T_x_ MXene acted as a conductive filler and, by forming a synergistic conductive network with LM and LiCl, collectively enhanced the hydrogel’s electrical conductivity, mechanical strength, and EMI shielding efficiency. Sima et al. [75] employed MXene to reinforce a TA-Fe_3_O_4_@MXene-PSBMA/AM (abbreviated as TFM) dual-network hydrogel synthesized from tannic acid (TA), Fe_3_O_4_ nanoparticles, SBMA, AM, and polyethylene glycol dimethacrylate (PEGDA). Specifically, the large specific surface area and abundant surface functional groups of MXene enabled the formation of dynamic cross-linking points with Fe_3_O_4_ nanoparticles and polymer chains, improving tensile and compressive strength. Its two-dimensional layered structure, combined with the porous structure of the hydrogel, promoted multiple reflections and absorption of electromagnetic waves, enhancing electromagnetic loss capability. Simultaneously, it synergized with PSBMA molecular chains to form a dual conductive network, significantly increasing the overall electrical conductivity. Xie et al. [76] enhanced a CS/P(AA-co-AM)/MXene@PEI/Fe^3+^+Cu^2+^ (abbreviated as CSPMXP) dual-network hydrogel—synthesized from polyethyleneimine (PEI), CS, AA, AM, and Fe^3+^/Cu^2+^ metal ions—using Ti_3_C_2_T_x_ MXene; the preparation process for MXP-x is shown in Figure 11. Specifically, MXene increased the “filler-matrix” interface, aiding in energy dissipation and thus improving toughness. The PEI coating on the MXene surface, possessing excellent water solubility, promoted the uniform dispersion of the modified MXene within the hydrogel matrix. Furthermore, PEI could form strong hydrogen bonds with both MXene and the hydrogel matrix, enhancing interfacial adhesion and reinforcing the MXene, which improved the strength and toughness of the hydrogel.

Leveraging their unique two-dimensional structure, excellent electrical conductivity, and tunable surface functional groups, MXene plays multiple roles in dual-network hydrogels. They not only enhance the mechanical properties and electrical conductivity of the hydrogels but also confer excellent strain sensing and EMI shielding performance. Although the improvement in electrical conductivity by MXene is less pronounced than that achieved by metal ions, their primary contribution lies in significantly enhancing the mechanical properties of the hydrogels.

## 4. Applications in Flexible Electronic Devices

### 4.1. Bodily Fluid Biomarker Sensors

Biosensors for bodily fluid biomarkers are analytical devices that utilize specific biological recognition elements combined with physical/chemical transducers to quantitatively detect disease-related markers in biological fluids (e.g., blood, sweat, urine) [78,79]. Their core function is to achieve highly sensitive and specific detection of key indicators—such as adenosine triphosphate (ATP), glucose, pH/lactate—for early disease diagnosis, prognosis assessment, or treatment efficacy monitoring [80]. To address the typically time-consuming nature of biomarker detection methods, Li et al. [81] developed an antifouling electrochemical biosensor based on a CS and DNA dual-network hydrogel via physical cross-linking for detecting ATP in complex biological fluids. This dual-network hydrogel, by expanding the electrode surface area and hydrophilicity and incorporating an aptamer specific for ATP recognition, enabled the accurate determination of ATP in human serum. The biosensor demonstrated a linear detection range for ATP from 0.1 pM to 1.0 μM, with a low detection limit (LOD) of 0.033 pM. The ATP detection results in human serum and cell lysates were comparable to those from ELISA kits, with recovery rates ranging between 92.2% and 103.6%.

**Figure 12:**
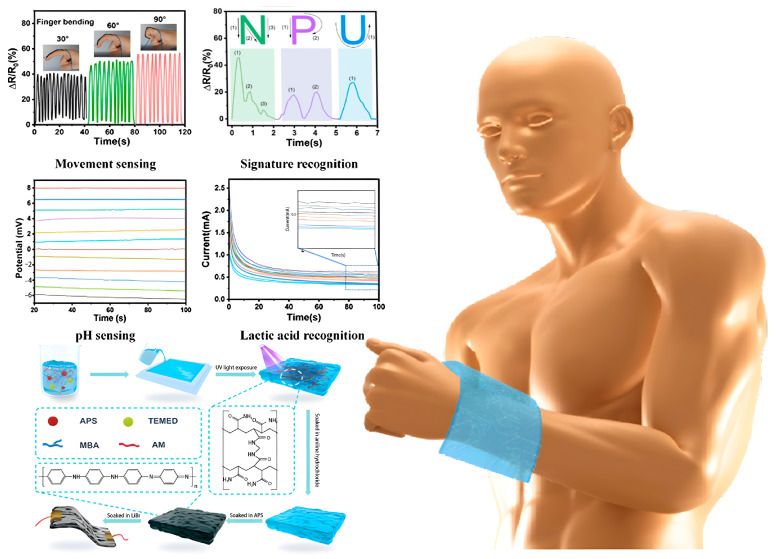
Application of dual-network hydrogels in bodily fluid biomarker sensors. Schematic diagram of the PAMAAni/LiBr hydrogel sensor for sweat detection [87]. Copyright 2025, Elsevier.

Conventional biomarker detection methods often require invasive sampling (e.g., blood draws) and rely on specialized equipment and personnel [82]. Sweat contains various biomarkers relevant to physiology and pathology, such as glucose and pH/lactate levels [83,84]. Dual-network hydrogels can be designed into wearable patch sensors that, through intimate skin contact, enable real-time and sensitive detection of biomarkers in sweat [85]. Shinde et al. [86] developed a wearable electrochemical sensor using a physico-chemically hybrid cross-linking method, featuring a DNH as an adhesive layer and a MoS_2_/PEDOT: PSS (MNP) nanocomposite as the coating electrode, for detecting glucose in sweat. The MNP-based electrode exhibited excellent electrochemical activity, with an electroactive surface area of 54.4 mm^2^, a glucose sensitivity of 9.76 μA·mM^−1^·cm^−2^, an LOD of 0.055 μM, and a detection range of 1–300 μM. Du et al. [87] constructed a dual-network hydrogel with excellent conductivity, low modulus, and remarkable anti-freezing capability via in situ photopolymerization. Flexible sensors assembled from this hydrogel demonstrated high performance in detecting sweat pH and lactate, as shown in Figure 12, specifically featuring a broad detection range (0.5–200%), high sensitivity (gauge factor = 3.24 within the 50–150% strain range), and excellent anti-fatigue performance (over 2000 cycles). This flexible sensor also performed outstandingly in electrochemical detection: it achieved a wide pH detection range of 1–12, a lactate detection range of 0.25–50 mM, and a low detection limit of 1.98 μM.

The application of dual-network hydrogel sensors in bodily fluid biomarker detection represents a technological breakthrough, addressing the invasiveness and time-consuming limitations of traditional methods through high adhesiveness, wide detection ranges, and low detection limits. Their optimized design (e.g., the dual-network structure) shows significant potential in wearable healthcare. Future research could explore their extensibility for simultaneous multi-marker detection or integrate wireless transmission modules to enable remote health monitoring [88,89].

### 4.2. Flexible Energy Storage Devices

**Figure 13:**
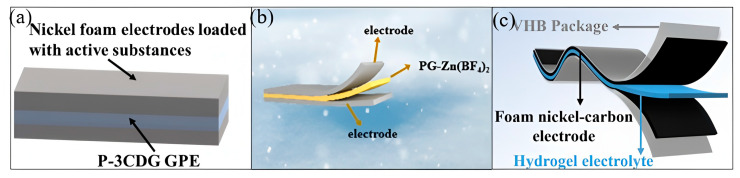
Application of dual-network hydrogels in flexible energy storage devices: (**a**) Schematic diagram of a capacitor assembled using P-3CDG GPE [92]. Copyright 2025, Elsevier. (**b**) Schematic diagram of the assembled supercapacitor device [95]. Copyright 2025, Elsevier. (**c**) Schematic diagram of supercapacitors based on PAM/PVA/LiTFSI-x% hydrogel [96]. Copyright 2024, Elsevier.

Flexible energy storage devices are bendable, stretchable, or twistable energy storage systems that provide power support for wearable electronics, flexible displays, and biomedical devices [90]. The core breakthrough in flexible energy storage lies in the innovation of electrolyte materials [91]. Che et al. [92] demonstrated a multifunctional polyvinyl alcohol–polyacrylic acid dual-network hydrogel (P-3CDG) via physico-chemical hybrid cross-linking, as shown in Figure 13a. When used as a gel polymer electrolyte (GPE), P-3CDG exhibited exceptional liquid absorption capacity (356.37 wt%) and high ionic conductivity (199.97 mS cm^−1^ after KOH absorption). In flexible supercapacitor applications [93], the hydrogel achieved a specific capacitance of 216.85 mF cm^−2^ at a current density of 1 mA cm^−2^, and an energy density of 30.12 Wh cm^−2^ at a power density of 195.11 W cm^−2^. Furthermore, zinc–air batteries (ZABs) assembled with 45% P-3CDG GPE [94] maintained excellent charge–discharge performance and high cycling stability after 147 cycles (49 h) at 1 mA cm^−2^, with an energy efficiency of 65.05%. Deng et al. [95] designed a dual-network hydrogel electrolyte (PGx-Zn) through physico-chemical hybrid cross-linking, as illustrated in Figure 13b. The PG3-Zn hydrogel (containing 3 wt% gelatin) exhibited a high fracture elongation of 1193% and a tensile stress of 88 kPa. At −80 °C, PG3-Zn showed an ionic conductivity of 0.26 S m^−1^, enabling the supercapacitor to retain 73%, 68%, and 61% of its room-temperature capacitance at −40 °C, −60 °C, and −80 °C, respectively. Moreover, the flexible supercapacitor showed negligible capacitance loss under bending deformations of 60°, 90°, and 180°. At −40 °C, it maintained a high capacitance retention of 90.3% and nearly 100% Coulombic efficiency after 5000 cycles. Dong et al. [96] developed a PAM/PVA/LiTFSI dual-network hydrogel via physical cross-linking for flexible energy storage devices, as depicted in Figure 13c. Supercapacitors employing the PAM/PVA/LiTFSI hydrogel as the electrolyte demonstrated an areal specific capacitance of 383.4 mF cm^−2^ at 0.5 mA cm^−2^, retained 90.35% of their initial capacitance after 10,000 cycles, and exhibited excellent resistance to bending deformation.

The common advantage of these three types of hydrogels lies in their enhanced mechanical strength and ion transport pathways enabled by the dual-network structure. The high conductivity of P-3CDG makes it suitable for high-power ZABs; the cryogenic tolerance of PGx-Zn extends applications to polar or aerospace environments; and the hydrogel developed by Dong’s team offers a long cycle life for daily wearable devices. These advances promote the development of flexible energy storage toward higher efficiency, durability, and environmental adaptability.

### 4.3. Health Monitoring Sensors

Health monitoring sensors are technologies integrated into wearable devices or medical instruments for the continuous, real-time detection and quantification of human physiological parameters, biochemical markers, or environmental factors to assess an individual’s health status. These sensors enable non-invasive collection of critical data, such as information related to flat feet, electrocardiograms, and respiration, thereby supporting early disease diagnosis, personalized health management, and telemedicine [97].

**Figure 14:**
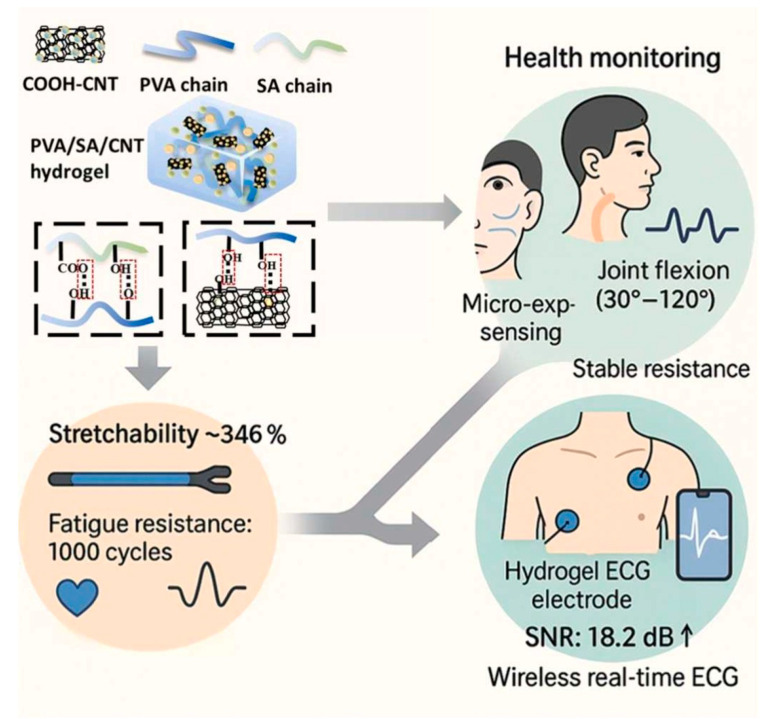
Schematic diagram of PVA/SA/CNT hydrogel detecting subtle facial movements, large joint flexion, and ECG signals [99]. Copyright 2025, Elsevier.

Ling et al. [98] employed chemical cross-linking to synthesize a conductive dual-network hydrogel (DNH) with excellent compressibility, good cyclic compression performance, repeatable adhesion, and high sensitivity for health monitoring. They integrated these sensors into 28 distinct zones of a custom insole. By measuring the resistance changes in the hydrogels in different areas under load, significant differences in plantar pressure distribution between normal feet and flat feet could be accurately identified. This study demonstrates that this research provides a novel and reliable material solution for the early screening, diagnosis, and evaluation of corrective effects for flat feet. Ding et al. [99] utilized physical cross-linking to synthesize a PVA/SA/CNT dual-network hydrogel with excellent conductivity, high stretchability, good elastic recovery, fatigue resistance, high sensitivity, and rapid response, applying it to health monitoring, as shown in Figure 14. The novel flexible ECG monitoring electrode based on this hydrogel, leveraging its superior flexibility, self-adhesiveness, and three-dimensional porous structure, forms tight conformal contact with the skin. During ECG monitoring, it significantly increased the signal-to-noise ratio from 7.5 dB (conventional electrodes) to 18.2 dB. During activities like walking and running, it effectively suppressed motion artifacts while still allowing clear identification of key waveform features such as P waves, QRS complexes, and T waves, overcoming the signal distortion issues of traditional electrodes in dynamic monitoring. Furthermore, the electrode demonstrated good breathability and biocompatibility, causing no skin irritation after 8 h of continuous wear, enabling comfortable and accurate long-term dynamic ECG monitoring. Wu et al. [100] synthesized a PVA/PAAM/CMC-Na/IL-COOH dual-network hydrogel (PPCMI) via physico-chemical cross-linking for health monitoring. A sensor developed from this hydrogel spontaneously generated an output voltage of 0.62 V at 43% RH, with a power density of 1.14 µW cm^−2^, and retained approximately 95% of its performance after 1000 bending cycles. A miniaturized sensor integrated into a mask could track breathing patterns in real-time by detecting humidity changes in exhaled breath, clearly distinguishing between different respiratory states for emotional state analysis. Specifically, by combining respiratory patterns with a one-dimensional convolutional neural network (1D-CNN) algorithm to identify the user’s emotion, the sensor achieved an overall recognition accuracy of 85.94% for three emotional states (calm, tense, and angry), with the highest accuracy of 98.75% for the “tense” emotion.

DNH constructed through various cross-linking strategies—chemical, physical, or physico-chemical—has emerged as an ideal material platform for developing advanced health monitoring sensors, owing to its tunable mechanical properties, reliable conductivity, good biocompatibility, and self-adhesiveness. Sensors based on these materials enable highly sensitive, rapid-response, and stable monitoring of various physiological signals (e.g., pressure, electrophysiological signals, and humidity), effectively suppressing motion interference and significantly enhancing signal quality. By integrating these sensors with intelligent algorithms, precise analysis ranging from basic physiological parameters to complex health states (such as emotion recognition) is achieved. This provides novel, reliable, and comfortable material solutions for dynamic health monitoring, early disease screening, diagnostic assessments, and personalized medicine.

### 4.4. Physical Motion Sensors

Physical motion sensors are a category of sensing devices specifically designed to detect the motion state parameters of objects (such as the human body), typically by measuring physical quantities like acceleration, angular velocity, position, or velocity, to capture dynamic change information [101].

**Figure 15:**
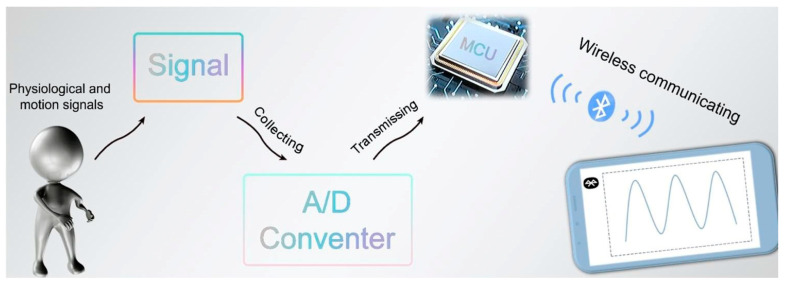
Application of dual-network hydrogels in physical motion sensors: Schematic diagram of an integrated sensing system for physiological and motion sensing signal acquisition and wireless transmission [102]. Copyright 2024, Elsevier.

Zheng et al. [102] developed a dynamic reversible dual-network hydrogel via a physico-chemical dual cross-linking strategy for physical motion sensors, as shown in Figure 15. This hydrogel exhibited high stretchability, tensile strength, compressive strength, and approximately 90.3% transparency. An integrated wireless wearable sensing system based on this hydrogel demonstrated exceptional long-term water retention capacity (over 2424 h with a retention rate exceeding 97%) and high sensitivity, enabling the real-time capture and transmission of physiological signals and motion information. Liu et al. [103] developed a dynamic physical/chemical dual cross-linked interpenetrating network hydrogel to enhance the performance of underwater physical motion sensors. The hydrogel exhibited high fracture strain, high mechanical strength, and a self-healing efficiency of 83%. Sensors based on this hydrogel demonstrated excellent underwater adhesion (65.4 kPa after 24 h of bonding in water) and anti-swelling capacity, providing high sensitivity (GF = 4.83) over a wide strain range (1–1000%), enabling accurate monitoring and signal transmission of human motion. Li et al. [104] designed a nanocomposite dual-network anisotropic hydrogel via physico-chemical hybrid cross-linking for application in physical motion sensors. This dual-network organohydrogel possessed excellent mechanical properties (tensile strength ~1.35 MPa, maximum fracture strain ~495%) and long-term water retention capacity (>24 h with a water retention rate > 97%). The hydrogel-based sensor could sensitively monitor human physiological signals and motions, such as precisely detecting finger joint bending from 15° to 90°, the direction and amplitude of wrist movement, and complex joint motions of the elbow and knee. The sensor exhibited rapid response and precise measurement under different stretching rates, maintaining stability after 2500 s of cyclic stretching. Pressure sensing tests revealed a response time of 336 ms and low hysteresis of 818 ms, enabling accurate identification of different pressure magnitudes and change rates. Furthermore, it was successfully applied in handwritten letter recognition (“U”, “S”, “T”, “C”) and full-body motion monitoring (e.g., walking, running, and jumping).

By modulating the cross-linking methods, composition, and structure of hydrogels, their properties can be precisely tailored to meet the requirements of different application scenarios. Integrating excellent mechanical properties (such as high stretchability and strength), long-term water retention capacity, and self-healing characteristics, DNHs serve as a foundation for wearable sensors that exhibit high sensitivity, rapid response, and outstanding stability. These sensors can accurately monitor a wide range of human activities, from subtle physiological signals to large-amplitude joint movements, and show promising application prospects in complex scenarios such as motion pattern recognition [105]. Table 4 shows the performance required for DNH to be applied in the field of flexible wearable electronics.

**Table 4:** Performance required for DNH applied in the field of flexible wearable electronics.

Performance Category	Specific Performance	References
Mechanical properties	Tensile strength/stress	[16]
	Self-recoverability	[22]
	Toughness	[24]
	Elongation at break/Tensile property/Ductility	[33]
	Fatigue resistance	[44]
	Compressive strength	[75]
Physical properties	Adsorption performance	[15]
	Adhesion	[20]
	Anti-swelling property	[26]
	Thermal responsiveness	[32]
	Humidity monitoring range	[38]
	Swelling behavior	[40]
	Electrical conductivity/Ionic conductivity	[43]
	Thermoelectric responsiveness	[45]
	Sensitivity	[53]
	Response time	[59]
	Low hysteresis energy	[59]
	Optical anisotropy	[60]
	Freeze resistance	[61]
	Stability	[66]
	Electromagnetic interference shielding efficiency	[74]
Structural characteristics	Homogeneity	[15]
	Interconnected Porous Structure	[21]
	Nanophase Separation	[34]
	Photonic Crystal Structure	[60]
	Conductive Pathways Formed by High-Aspect-Ratio Fillers	[73]
Other characteristics	Environmental adaptability	[20]
	pH responsiveness	[34]
	Biocompatibility	[39]

## 5. Conclusions and Challenges

DNH, as a functional material composed of two interpenetrating polymer networks, exhibits remarkable mechanical properties and functional diversity. Their preparation methods include physical crosslinking, chemical crosslinking, and physicochemical crosslinking, with each method endowing DNH with distinct characteristics. The incorporation of modified fillers, such as nanocellulose, carbon nanotubes, and MXene, can mitigate the defects caused by the crosslinking methods. Additionally, the applications of DNH in the field of flexible electronics have been reviewed. To date, extensive research has been conducted on DNH, leading to significant advancements, though certain challenges still remain.

(1) Weak anti-interference capability: Although high-sensitivity detection of biomarkers such as ATP and glucose can be achieved (e.g., the detection limit for ATP is 0.033 pM), the presence of interfering components like proteins and salts in complex bodily fluids (e.g., serum, sweat) can easily cause non-specific binding with the hydrogel network, leading to detection deviations. Existing antifouling designs (e.g., DNA modification) are tailored only for single biomarkers, making it difficult to adapt to simultaneous multi-biomarker detection.
(2) Accelerated performance degradation due to environmental aging: When hydrogels are exposed to light and oxygen for extended periods, polymer chain degradation (e.g., PVA hydrolysis, MXene oxidation) is prone to occur, resulting in a 30–50% decline in mechanical properties and conductivity within 6 months. Moreover, in high-temperature (>40 °C) and high-humidity (relative humidity > 80%) environments, hydrogels are susceptible to “swelling-shrinking” cycles, gradually damaging the network structure and shortening their service life.
(3) Complex preparation processes, high costs, and challenges in mass production: Most existing high-performance hydrogels rely on precisely controlled laboratory processes, such as “freezing-assisted metal complexation” and “insitu photopolymerization-freeze-thaw cycling.” Additionally, hydrogels from different batches fail to meet the “consistency” requirements of industrial production. The preparation costs of modified fillers (e.g., MXene, aminated CNTs) are high, and some processes depend on rare reagents (e.g., ionic liquids, specific enzymes). Furthermore, the storage of hydrogels requires sealed, low-temperature environments, further increasing warehousing and transportation costs.

## 6. Future Perspectives

In the future, it is necessary to explore new types of hydrogel materials that are convenient, have strong anti-interference ability and a low cost, and can be used for a long time for wearable sensors.

In addition, current research endeavors are focused on developing smart hydrogels responsive to multiple stimuli (such as temperature, humidity, light, or pressure), which could be further applied in complex health monitoring and human–computer interaction systems. For instance, dual-responsive hydrogels demonstrate unique advantages in simulating dynamic alterations within complex biological environments. In fields such as chronic disease monitoring, tissue repair, or soft robotics, future work could further integrate sensing technologies with the biochemical compatibility and dynamic self-healing properties of functional hydrogels. Continuously improving the interactivity and durability of hydrogels within biological environments remains an important research direction.

## Data Availability

No new data were created or analyzed in this study. Data sharing is not applicable to this article.
